# Modulation of LSD1 phosphorylation by CK2/WIP1 regulates RNF168-dependent 53BP1 recruitment in response to DNA damage

**DOI:** 10.1093/nar/gkv528

**Published:** 2015-05-20

**Authors:** Bin Peng, Jing Wang, Yuan Hu, Hongli Zhao, Wenya Hou, Hongchang Zhao, Hailong Wang, Ji Liao, Xingzhi Xu

**Affiliations:** Beijing Key Laboratory of DNA Damage Response and College of Life Sciences, Capital Normal University, Beijing 100048, China

## Abstract

Proper DNA damage response is essential for the maintenance of genome integrity. The E3 ligase RNF168 deficiency fully prevents both the initial recruitment and retention of 53BP1 at sites of DNA damage. In response to DNA damage, RNF168-dependent recruitment of the lysine-specific demethylase LSD1 to the site of DNA damage promotes local H3K4me2 demethylation and ubiquitination of H2A/H2AX, facilitating 53BP1 recruitment to sites of DNA damage. Alternatively, RNF168-mediated K63-linked ubiquitylation of 53BP1 is required for the initial recruitment of 53BP1 to sites of DNA damage and for its function in repair. We demonstrated here that phosphorylation and dephosphorylation of LSD1 at S131 and S137 was mediated by casein kinase 2 (CK2) and wild-type p53-induced phosphatase 1 (WIP1), respectively. LSD1, RNF168 and 53BP1 interacted with each other directly. CK2-mediated phosphorylation of LSD1 exhibited no impact on its interaction with 53BP1, but promoted its interaction with RNF168 and RNF168-dependent 53BP1 ubiquitination and subsequent recruitment to the DNA damage sites. Furthermore, overexpression of phosphorylation-defective mutants failed to restore LSD1 depletion-induced cellular sensitivity to DNA damage. Taken together, our results suggest that LSD1 phosphorylation modulated by CK2/WIP1 regulates RNF168-dependent 53BP1 recruitment directly in response to DNA damage and cellular sensitivity to DNA damaging agents.

## INTRODUCTION

Genomic DNA damage induced by environmental agents and cellular intermediate metabolites constantly occurs. Timely and proper response to such damage protects genome integrity and prevents tumorigenesis. Among many forms of DNA damage, DNA double-strand breaks (DSBs) are thought to be the most harmful threat to genome stability and cell survival since a single unrepaired DSB is sufficient to induce cell death. The overall DNA damage response (DDR) is governed by coupling protein–protein interaction networks and post-translational modifications, among which phosphorylation and ubiquitination are well documented (1,2). The DSB signal is recognized and transduced by the central ATM (ataxia-Telangiectasia mutated)-CHK2 (checkpoint kinase 2) kinase cascade, whose activation is facilitated by a variety of mediators, including the MRN (MRE11-RAD50-NBS1) complex, MDC1 (mediator of DNA damage checkpoint 1) and 53BP1 (p53 binding protein 1). MDC1-dependent recruitment of the Ring finger E3 ligases RNF8-RNF168 is the major early ubiquitination event, which ubiquitinates H2A/H2AX and many other substrates and facilitates DSB signaling and repair (3–5). A DSB is mainly repaired by homologous recombination with high fidelity or by non-homologous end-joining (NHEJ) which is error prone. Several DDR players, including 53BP1, the telomere-associated protein RIF1 (Rap1-interacting factor 1 homolog) and PTIP (PAX-interacting protein 1), have been implicated in dictating the DSB repair pathway choice (6–11).

53BP1 is an important DDR player implicated in DSB checkpoint signaling, repair pathway choice and NHEJ-mediated DSB repair (6). Its recruitment to the DNA damage site has been thought to be dependent largely on two histone modifications, namely H4K20me2 and H2AK15ub. The tandem Tudor domains of 53BP1 specifically bind to H4K20m2 but are not sufficient for its accumulation, while its ubiquitination-dependent recruitment motif recognizes the H2A ubiquitylation at K15, which is catalyzed by RNF168, to sustain the stable recruitment of 53BP1 (6,12,13). 53BP1 dimerization further promotes its recruitment (14). It has been reported that the methyltransferases SET8, SUV420 and MMSET target H4K20 locally for mono- and dimethylation at damage sites, while RNF8/RNF168-dependent ubiquitination and subsequent proteasome-mediated degradation of the histone demethylase KDM4A in response to DNA damage exposes H4K20me2, which is bound and thus masked by the Tudor domain of KDM4A under unperturbed conditions (6,15–17). In addition, the AAA-ATPase VCP/p97 (valosin-containing protein)-containing complex is recruited to DSBs, displaces the Polycomb group protein L3MBTL1 (Lethal(3) malignant brain tumor-like protein 1) from chromatin and exposes H4K20me2 (18). These joint actions unmask H4K20me2 on the chromatin flanking the DNA damage site for 53BP1 binding. Furthermore, DNA damage-induced localized deacetylation of H4K16 and H3K56 by histione deacetylase 1/2 (HDAC1/2) makes H4K20me2 more accessible for 53BP1 (19,20). The E3 ligase RNF168 deficiency is linked to the human radiosensitivity, immunodeficiency, dysmorphic features and learning difficulties syndrome (21). This syndrome is at least partially explained by the fact that RNF168 deficiency fully prevents both the initial recruitment to and retention of 53BP1 at sites of DNA damage (21,22). In response to DNA damage, RNF8-independent but RNF168-dependent recruitment of LSD1 (Lysine-specific demethylase-1) to the site of DNA damage promotes local H3K4me2 demethylation and ubiquitination of H2A/H2AX, facilitating 53BP1 recruitment to the site of DNA damage (23). LSD1 is a member of the FAD-dependent amine oxidase demethylase family. It acts as an ‘eraser’ to remove methyl group from mono- or dimethylated H3K4 (24). It was recently also shown that RNF168-mediated K63-linked ubiquitylation of 53BP1 is required for the initial recruitment of 53BP1 to sites of DSBs and for its function in DNA damage repair (22).

Casein kinase 2 (CK2) is a constitutively active serine/threonine kinase that is ubiquitously expressed. Its tetrameric holoenzyme is typically composed of two regulatory β subunits and two catalytic subunits (α and α’). Although CK2α shares 90% sequence identity to CK2α’, CK2α knockout mice are not viable while CK2α’ knockout mice are viable, indicating that CK2α is not completely functionally redundant with CK2α’ (25–27). CK2 possesses hundreds of substrates (http://www.phosphosite.org) implicated in many cellular activities, including cell proliferation and survival, cell cycle progression and genotoxic stress response (28,29). CK2-mediated phosphorylation of the SDT repeats in the amino-terminus of MDC1 recruits NBS1 and, thereby, increases the local concentration of MRN at the damaged DNA sites (30). Inhibition of CK2 expression decreases the binding of 53BP1 to H4K20me2 and 53BP1 recruitment to the DNA damage site (31). However, the molecular mechanism of CK2-mediated recruitment of 53BP1 to the sites of DNA damage is not fully understood.

Wild-type p53-induced phosphatase 1 (WIP1, also known as PPM1D) is a serine/threonine phosphatase of the PP2C family (32). Unlike members of PP2A or PP1 family, WIP1 acts as a monomer, and its catalytic activity is dependents on Mg^2+^ or Mn^2+^ and is insensitive to inhibition by okadaic acid or microcystin (33,34). A number of DDR factors have been demonstrated to be substrates of WIP1, including ATM (S367, S1981), γ-H2AX, CHK1 (S345) and CHK2 (T68) (34–38).

In this report, we uncovered that RNF168, LSD1 and 53BP1 interact with each other directly and that CK2/WIP1-mediated modulation of LSD1 phosphorylation at S131 and S137 facilitates RNF168-dependent ubiquitination and recruitment of 53BP1 to the DNA damage sites, a process which promotes cell proliferation and survival in response to DNA damage.

## MATERIALS AND METHODS

### Cell cultures, reagents, antibodies and expression constructs

HeLa, U2OS, 293T and HEK293 cells were cultured in high-glucose Dulbecco's modified Eagle's medium supplemented with 10% FBS (vol/vol, Biological Industries, 1347477) at 37°C. Bleomycin was purchased from Nippon Kayaku Co, etoposide (E1383) and TBB (T0826) were from Sigma, respectively. GST-CK2α (10630-H09B) and His-GST CK2α’ (11018-H20B) produced in and purified from insect cells were purchased from Sino Biological Inc.

Rabbit polyclonal antibodies used in this study including anti-HA (A190–208A), anti-MYC (A190–205A), anti-LSD1 (A300–215A), anti-53BP1 (A300–272A), anti-CK2α (A300–198A), anti-CK2α’ (A300–199A), anti-CK2β (A301–984A), anti-GAPDH (A300–643A) and anti-WIP1 (A300–664A) were purchased from Bethyl, while rabbit anti-RNF168 (06–1130) polyclonal antibodies were from Millipore. Mouse monoclonal antibodies including anti-FLAG M2 (F1804) and anti-β-actin (A5441) were from Sigma. Rabbit phospho-sepcific antibodies against LSD1(S131) and LSD1(S137) were generated and affinity-purified by AbMart and Beijing B&M Biotech using the phosphopeptides SLANL (pS) EDEYY and CDEYY (pS) EEERN, respectively.

Human cDNA clones encoding LSD1, 53BP1, 53BP1-KBD (residues 1234–1616), RNF168, WIP1, CK2α, CK2α’ and CK2β were subcloned into pcDNA3.0 with three copies of HA or FLAG epitope at its N-terminus or one copy of MYC epitope at its C-terminus. Point mutants were generated using the QuikChange mutagenesis kit (Stratagene). LSD1 rescue constructs were generated by silent mutation of 10 nucleotides within the shLSD1–1-targeting region. The expression construct of HA-UB(K63), in which only the K63 was wild-type while all the other lysine residues were mutated into arginine, was described before (39). All the expression constructs were confirmed by DNA sequencing. Detailed cloning information is available upon request.

### RNA interference

The siRNA oligonucleotide duplexes targeting CK2α (sequence: GAUGACUACCAG CUGGUUCdTdT), CK2α’ (sequence: CAGUCUGAGGAGCCGCGAGdTdT), LSD1 3′ UTR (sequence: GCUCUUCUAGCAAUACUAGdTdT) or RNF168 (siRNA#1: CTTTAAAGATGCAGTTGAA; siRNA#2: GTGGAACTGTGGACGATAA; siRNA #3: GGAACTGAGAAGAGAATAT) were purchased from Guangzhou Ribo-bio. Control siRNA oligonucleotide duplex (siCTR sequence: CGUACGCGGAAUACUU CGAdTdT) was purchased from Shanghai GenePharma. Cells were transfected with a siRNA complex at a final concentration of 20 nM using RNAiMAX transfection reagent (Invitrogen). The shCTR and sh-LSD1 (shLSD1–1: CACCGCCTGTTTCTGC CATGTAAGGCGAACCTTACATGGCAGAAACAGGC, shLSD1–2: CACCGCCAT GTAAGGAAGGCTCTTCCGAAGAAGAGCCTTCCTTACATGGC, shLSD1–3: CA CCGGAAGGCTCTTCTAGCAATACCGAAGTATTGCTAGAAGAGCCTTCC) lentiviral particles were purchased from Shanghai Novobio.

### Generation of HEK293 cells stably expressing HA-LSD1 or its point mutants by retroviral infection

The coding region of wild-type LSD1 or its point mutants (LSD1 (S131A), LSD1 (S137A), LSD1 (S2A) and LSD1 (K661A)) was subcloned into the retroviral vector pBabe-puro with an N-terminal HA tag. The retroviral constructs were co-transfected with Gag-Pol and V-SVG into 293T cells. Medium was collected 48 h after transfection, filtered through a microfilter with a pore size of 0.45 μm and subsequently used to infect HEK293 cells in the presence of polybrene. Infectants were cultured in the medium in the presence of puromycin at a final concentration of 1 μg/ml for 1 week. The survived cell population was used for subsequent experiments.

### Immunoprecipitation, immunoblotting and immunofluorescence staining

Immunoprecipitation and/or immunoblotting were performed as described before (40). For the indirect immunofluorescence staining, U2OS cells cultured on coverslips were washed once with PBS, fixed with ice-cold methanol for 5 min and blocked with 2% BSA solution in PBST (0.1% tween-20 in PBS) for 30 min. These cells were incubated with appropriate primary antibody for 60 min, washed three times with PBST and incubated with fluorescence-conjugated secondary antibody for 60 min. The dilutions for the anti-53BP1 and anti-FLAG antibodies were 1:500 and 1:1000, respectively. After extensive washing with PBST, cells were stained with 4,6-diamidino-2-phenylindole for 2 min. The coverslips were mounted onto glass slides with an anti-fade solution and visualized using an Olympus IX-81 fluorescence microscope.

### Laser microirradiation-induced DNA damage

U2OS cells grown on a dish with thin glass bottom were locally irradiated with a 365 nm pulsed nitrogen UV laser (16 Hz pulse, 41% laser output) generated from the micropoint system (Andor). This system was directly coupled to the epifluorescence path of the Nikon A1 confocal imaging system with time-lapse imaging every 30 s for 10 min.

### GST pulldown assays and *in vitro* kinase assays

Bacterially-produced or insect cells-produced GST fusions (1 μg) were incubated with bacterially produced HIS-tagged fusions (1 μg) in 500 μl of NETN buffer (20 mM Tris-HCl [pH 8.0], 0.15 M NaCl, 1 mM EDTA, 0.5% NP-40 and protease inhibitor cocktail) at 4°C overnight. Glutathione-Sepharose beads (20 μl per pulldown) were added and incubated for 1 h before extensively washing with NETN buffer.

*In vitro* kinase assays were performed as described before (41) except that the CK2 kinase buffer contained 50 mM HEPES (pH 7.4), 10 mM MgCl2, 1 mM DTT, 1 mM Na_3_VO_4_, 10 μM cold ATP and 5 μCi [γ-^32^P]-ATP. The GST pulldown after kinase assay was performed by adding 500 μl of NETN buffer into the reaction after the *in vitro* kinase assay was completed.

### *In vitro* dephosphorylation assay

The endogenous LSD1 immunocomplex and the HA-LSD1 immunocomplex were prepared from 293T cells and 293T cells transiently expressing HA-LSD1, respectively. For WIP1-mediated dephosphorylation assay *in vitro*, the immunocomplexes were washed once with phosphatase buffer (50 mM Tris-HCl (pH 7.5), 30 mM MgCl_2_, 1 mg/ml bovine serum albumin, 0.05% 2-mercaptoethanol), and incubated with bacterially produced GST, GST-WIP1 or GST-WIP1 (D314A) at 30°C for 1 h, and the reaction was stopped by adding sodium dodecyl sulphate sample buffer (34). For calf intestine phosphatase (CIP, M0290, New England Biolabs) treatment, the immunocomplexes were washed and incubated with NEB buffer 3 instead.

### Cell synchronization

U2OS cells were synchronized at late S/G2 phase by double thymidine block and release as described before (40).

### Immunoprecipitation from the chromatin-enriched fraction

Chromatin fractionation assays were conducted essentially as described (40). The isolated chromatin-enriched fraction P3 was incubated with 500 μl of NETN at 4°C for 30 min and then centrifuged at 18 000 x *g* at 4°C for 10 min. The supernatants were processed for immunoprecipitation with an appropriate antibody.

## RESULTS

### CK2 phosphorylates LSD1 both *in vitro* and *in vivo*

**Figure 1.**
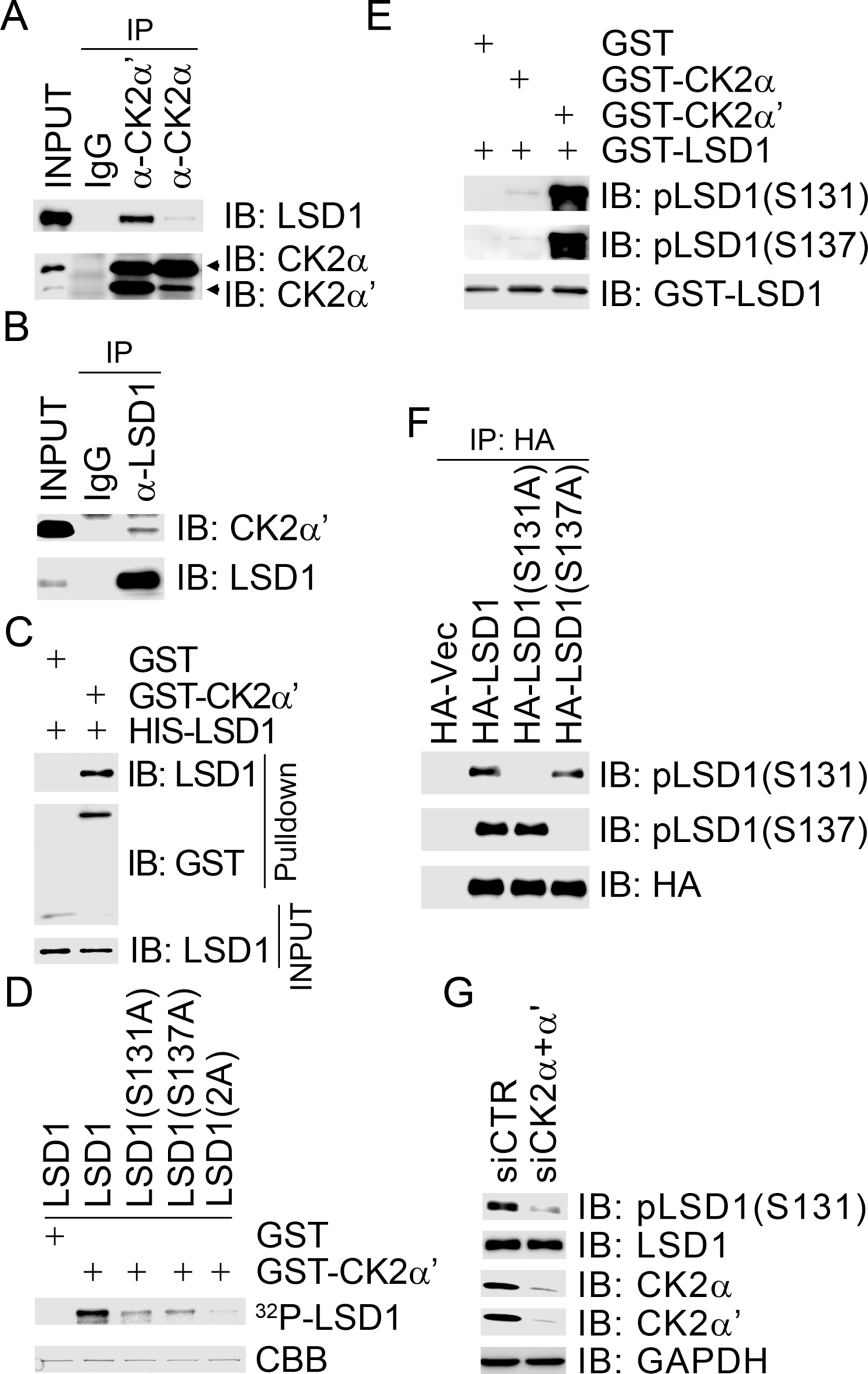
CK2 phosphorylates LSD1 at S131 and S137 both *in vitro* and *in vivo*. (**A** and **B**) Endogenous CK2α’ co-immunoprecipitated reciprocally with endogenous LSD1. Total cell lysates were extracted from 293T cells and subjected to immunoprecipitation followed by immunoblotting with antibodies as indicated. (**C**) LSD1 directly interacted with CK2α’. GST or GST- CK2α’ recombinant protein was employed to pull down bacterially produced HIS-LSD1. (**D**) CK2 phosphorylated LSD1 *in vitro*. GST or GST-CK2α’ recombinant protein was incubated with bacterially produced GST-LSD1 or its phosphorylation-defective mutants in the presence of ^32^P-γATP in the *in vitro* kinase assay. (**E**) The phospho-specific antibodies were reactive toward LSD1 phosphorylated by CK2 *in vitro*. Bacterially produced GST-LSD1 was incubated with GST-CK2 and subjected to immunoblotting with antibodies as indicated. (**F**) Specificity of the LSD1 phospho-specific antibodies. Total cell lysates were extracted from 293T cells transiently transfected with HA-Vec or expression constructs as indicated, and the HA immunocomplexes were then subjected to immunoblotting with antibodies as indicated. (**G**) Inhibition of CK2 expression diminished LSD1 phosphorylation at S131. Total cell lysates were extracted from HeLa cells with mock depletion or depletion of both CK2α and CK2α’ and subjected to immunoblotting with antibodies as indicated.

LSD1 undergoes phosphorylation modification during the cell cycle in unperturbed conditions (42). However, it is still largely unknown which kinase(s)/phosphatase(s) modulates LSD1 phosphorylation status. Recently, it has been demonstrated that epitope-tagged LSD1 physically interacts with endogenous CK2α *in vivo* and is potentially phosphorylated by CK2α on three serine residues (S131, S137, S166) *in vitro* by mass spectrometric analysis (43). These three potential phosphorylation sites reside within the N-terminus of LSD1, which has been believed not to contribute much to its catalytic activity (44). Consistent with these findings, we confirmed that both HA-tagged CK2α and CK2α’ physically associated with FLAG-tagged LSD1 (Supplementary Figure S1A and B). We further found that endogenous LSD1 in 293T cells co-immunoprecipitated with endogenous CK2α’, and with CK2α to a lesser extent (Figure 1A), and that endogenous CK2α’ was present in the endogenous LSD1 immunocomplex (Figure 1B). GST-CK2α’ generated and purified from insect cells was able to pull down bacterially produced HIS-LSD1 (Figure 1C), indicating that the interaction between LSD1 and CK2 is direct. *In vitro* kinase assays revealed that GST-CK2α’ efficiently phosphorylated wild-type LSD1 and point mutant LSD1(S166A), but not the point mutants LSD1(S131A) or LSD1(S137A) (Figure 1D and Supplementary Figure S1C), indicating that S131 and S137 are the major phosphorylation sites within LSD1 by CK2α’.

To further determine if LSD1 is phosphorylated at S131 and S137 *in vivo*, we raised two rabbit polyclonal antibodies against a pS131 phosphopeptide and a pS137 phosphopeptide, respectively, within the N-terminus of LSD1. Both antibodies were reactive to LSD1 recombinant protein in immunoblotting, which was incubated with GST-CK2α’ in an *in vitro* kinase assay (Figure 1E), and endogenous or HA-tagged LSD1 immunoprecipitated from 293T cells (Supplementary Figure S1D and E), whereas the specific signal disappeared after the LSD1 immunocomplex was treated with the CIP (Supplementary Figure S1D and E). Furthermore, the pLSD1(S131) antibody recognized both HA-LSD1 and HA-LSD1(S137A), but not HA-LSD1(S131A), while the pLSD1(S137) antibody was reactive to both HA-LSD1 and HA-LSD1(S131A), but not HA-LSD1(S137A) (Figure 1F). Inhibition of CK2a and CK2α’ expression in 293T cells greatly reduced pLSD1(S131)-specific signal (Figure 1G). Conversely, overexpression of CK2α’ in 293T cells slightly enhanced pLSD1(S131) and pLSD1(S137) signal under unperturbed conditions (Supplementary Figure S1F). Taken together, our data demonstrated that the phosphorylation-specific antibodies we generated, pLSD1(S131) and pLSD1(S137), are specific for detecting LSD1 phosphorylated at S131 and S137, respectively. Our results revealed that LSD1 is a physiological substrate of CK2 under unperturbed conditions.

### WIP1 interacts and dephosphorylates LSD1 both *in vitro* and *in vivo*

**Figure 2.**
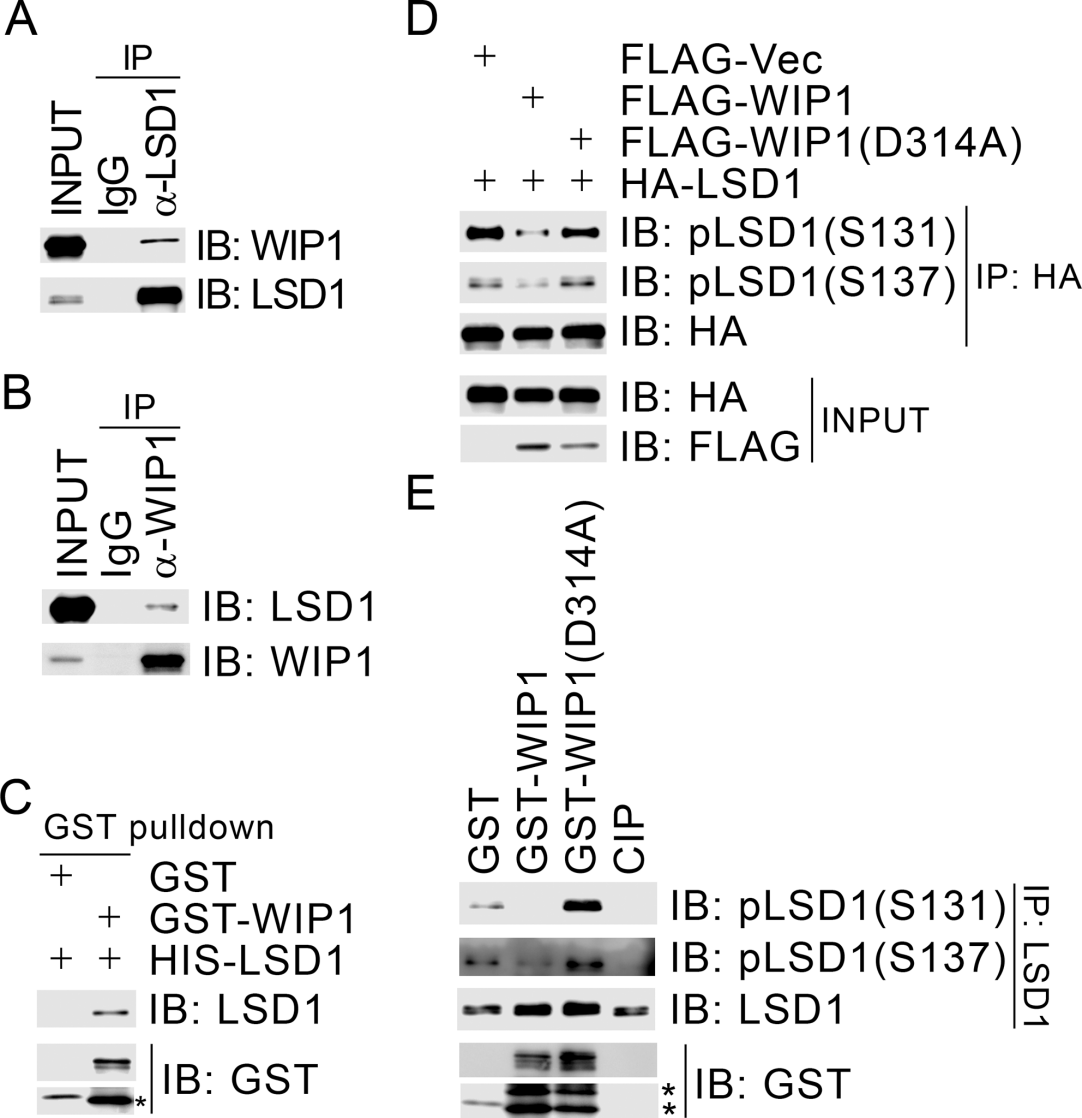
WIP1 dephosphorylates LSD1 at S131 and S137 both *in vitro* and *in vivo*. (**A** and **B**) Endogenous WIP1 co-immunoprecipitated reciprocally with endogenous LSD1. Total cell lysates were extracted from 293T cells and subjected to immunoprecipitation followed by immunoblotting with antibodies as indicated. (**C**) WIP1 directly interacted with LSD1. Bacterially produced GST-WIP1 was used to pull down bacterially produced HIS-LSD1 *in vitro*. (**D**) Overexpression of WIP1 decreased LSD1 phosphorylation at S131 and S137 *in vivo*. 293T cells were transiently transfected with expression constructs as indicated. Total cell lysates were harvested 48 h after transfection and subjected to immunoprecipitation with an anti-HA antibody followed by immunoblotting with antibodies as indicated. (**E**) WIP1 dephosphorylated LSD1 at S131 and S137 *in vitro*. CIP or bacterially produced GST, GST-WIP1 or GST-WIP1 (D314A) was incubated with the LSD1 immunocomplexes prepared from 293T cells in the *in vitro* dephosphorylation assay. *degraded GST fusions.

Our mass spectrometric analysis of the WIP1 immunocomplex from HeLa cells uncovered that HDAC1, CoREST and PF21A, components of a BRAF–HDAC complex (BHC) histone deacetylase complex, were present in the immunocomplex (data not shown). It has been known that LSD1 is also a component of the BHC complex (45). We thus wanted to determine if WIP1 interacts with LSD1. It was found that endogenous WIP1 was present in LSD1 immunocomplex (Figure 2A), and endogenous LSD1 was present in the WIP1 immunocomplex in 293T cells (Figure 2B). To exclude the possibility of a false-positive interaction resulting from cross-reactivity of antibodies, we found that FLAG-WIP1 physically associated with HA-LSD1 in 293T cells in the reciprocal co-immunoprecipitation assays (Supplementary Figure S2A and B). Furthermore, when bacterially produced GST-WIP1 and HIS-LSD1 were mixed together, GST-WIP1 specially pulled down HIS-LSD1 (Figure 2C), indicating that LSD1 directly interacts with WIP1.

We next examined if LSD1 is a substrate of WIP1. Expression of FLAG-WIP1 reduced phosphorylation levels of LSD1(S131) in a dose-dependent manner, while expression of the phosphatase-dead mutant WIP1 (D314A) did not have any impact on LSD1 phosphorylation (Figure 2D and Supplementary Figure S2C). In the *in vitro* dephosphorylation assays, bacterially produced wild-type GST-WIP1, but not GST-WIP1 (D314A), readily dephosphorylated S131- and S137-phosphorylated forms of LSD1 or HA-LSD1 enriched by immunoprecipitation from 293T cells (Figure 2E and Supplementary Figure S2D). These results demonstrated that WIP1 dephosphorylates LSD1 both *in vitro* and *in vivo*, suggesting that LSD1 is a physiological substrate of WIP1.

### CK2-mediated phosphorylation of LSD1 promotes its recruitment to sites of DNA damage

**Figure 3.**
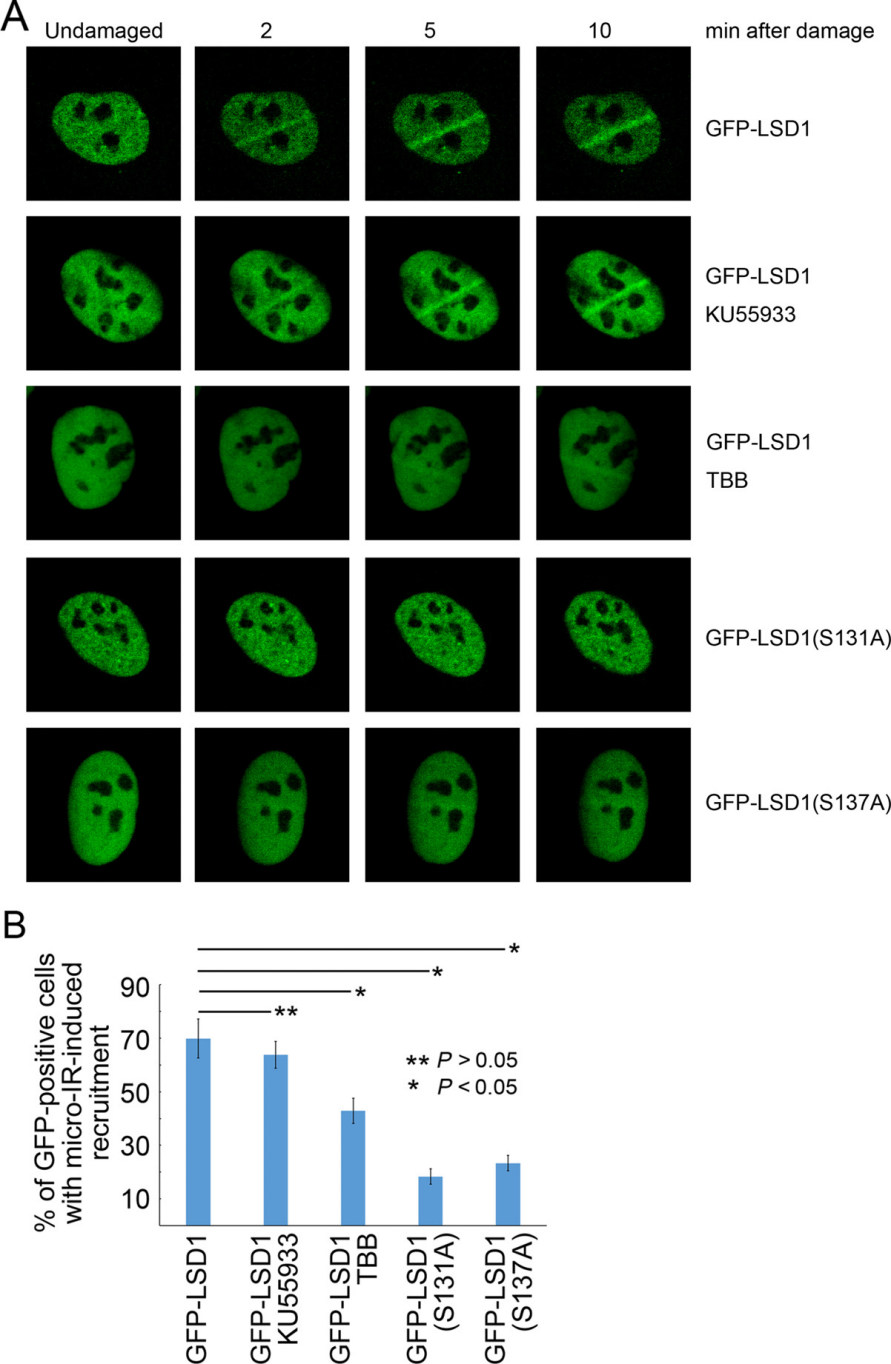
CK2-mediated phosphorylation of LSD1 promotes its recruitment to sites of DNA damage. U2OS cells transiently expressing GFP-LSD1, GFP-LSD1(S131A) or GFP-LSD1(S137A) were irradiated with a 365-nm UV laser beam, while some GFP-LSD1 expressing cells were also pretreated with KU55933 or TBB before irradiation. Images were collected every 60 s after irradiation. Representative images are shown in (**A**). In each set of experiment, more than 20 GFP-positive cells were irradiated and monitored for enrichment of GFP signal along the irradiation path. Three experimental repeats were performed and statistical analysis is shown in (**B**).

It has been reported that LSD1 is recruited to DNA damage sites within 10 min after irradiation (23). We therefore wished to determine if CK2-mediated phosphorylation of LSD1 is important for its concentration at sites of DNA damage induced by UV laser microirradiation. As shown in Figure 3, GFP-LSD1 in U2OS cells was obviously concentrated on the laser path as early as 3 min after irradiation (∼70% of irradiated GFP-positive cells). Treatment of cells with the ATM-specific inhibitor KU55933 did not significantly compromise LSD1 recruitment, while CK2-specific inhibitor TBB significantly reduced the LSD1 recruitment to the microirradiation path in GFP-LSD1-positive cells to 43%. More strikingly, both the phosphorylation-defective mutants GFP-LSD1(S131A) and GFP-LSD1(S137A) failed to be enriched at sites of DNA damage. On the other hand, overexpression of wild-type WIP1, but not the catalytically inactive mutant WIP1 (D314A) in U2OS cells, compromised micro-irradiation-induced recruitment of LSD1 (Supplementary Figure S3). These results demonstrated that CK2, and potentially other unidentified kinase(s)-mediated phosphorylation of LSD1 at S131 and S137, is important for its recruitment to sites of DNA damage.

### CK2-mediated phosphorylation of LSD1 promotes its binding to RNF168

**Figure 4.**
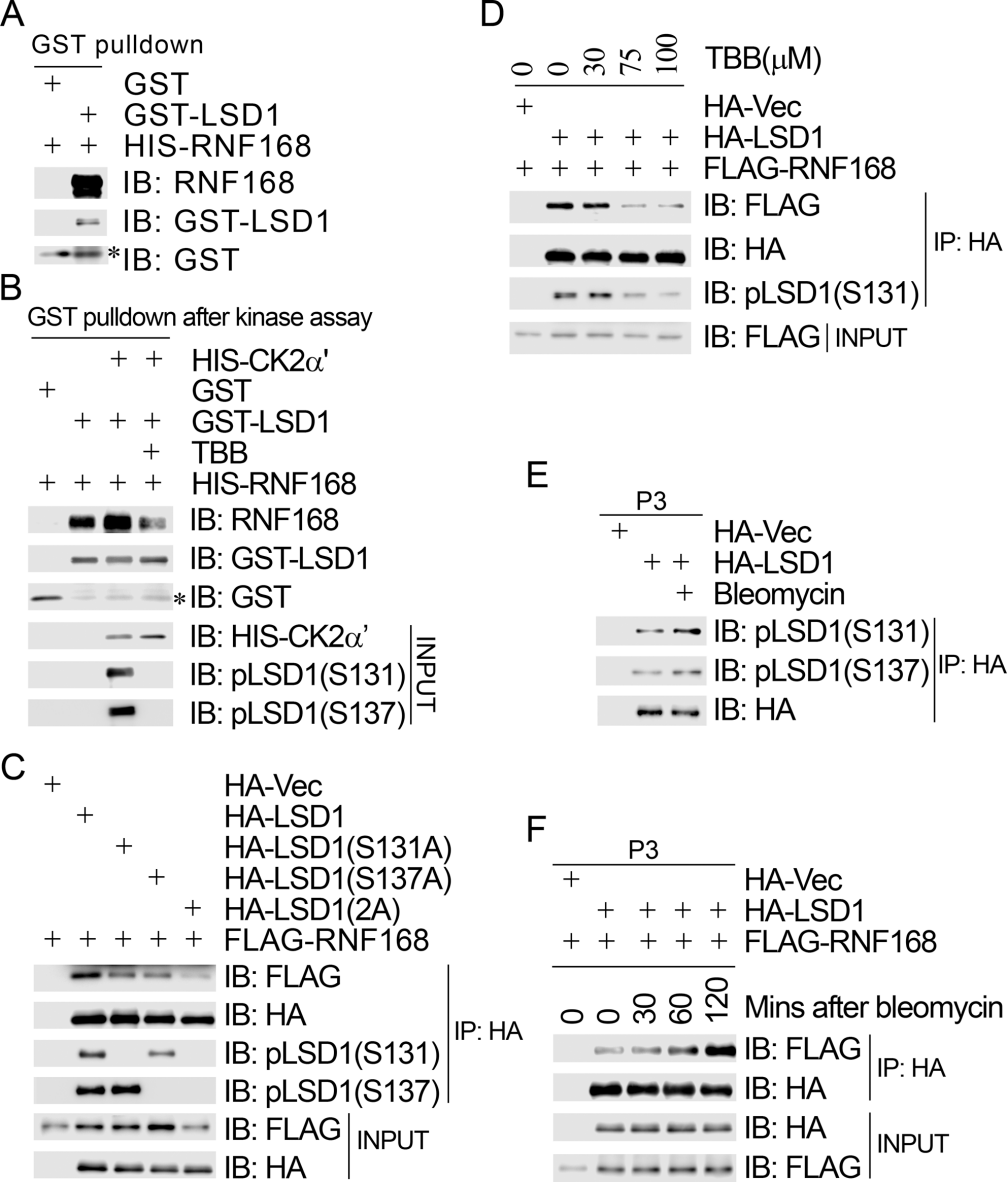
CK2-mediated phosphorylation of LSD1 promotes its association with RNF168. (**A**) LSD1 directly interacted with RNF168. Bacterially produced LSD1 was used to pull down bacterially produced HIS-RNF168 *in vitro*. (**B**) CK2-mediated phosphorylation of LSD1 enhanced its binding to RNF168 *in vitro*. Bacterially produced GST, GST-LSD1 and GST-LSD1 phosphorylated by CK2 *in vitro* in the absence/presence of the CK2 inhibitor TBB were employed to pull down bacterially produced HIS-RNF168. (**C**) Phosphorylation-defective mutants of LSD1 reduced their binding to RNF168 *in vivo*. 293T cells were transiently co-transfected with FLAG-RNF168 and HA-Vec/HA-LSD1/HA-LSD1(S131A)/HA-LSD1(S137A)/HA-LSD1(2A). Total cell lysates were extracted 48 h after transfection and subjected to immunoprecipitation with an anti-HA antibody followed by immunoblotting with antibodies as indicated. (**D**) Inhibition of CK2 activity *in vivo* reduced the binding of LSD1 to RNF168. 293T cells were co-transfected with FLAG-RNF168 and HA-Vec/HA-LSD1. Transfectants were treated with different concentrations of TBB 6 h before harvest. Total cell lysates were harvested 48 h after transfection and subjected to immunoprecipitation with an anti-HA antibody followed by immunoblotting with antibodies as indicated. (**E**) Bleomycin treatment increased LSD1 phosphorylation in the chromatin-enriched fraction. 293T cells were transiently transfected with HA-Vec or HA-LSD1. HA-LSD1 transfectants were either mock-treated or treated with bleomycin (10 μg/ml) for 1 h before harvest. Chromatin-enriched fractions (P3) were prepared 48 h after transfection and subjected to immunoprecipitation with an anti-HA antibody followed by immunoblotting with antibodies as indicated. (**F**) DNA damage induced an increase of interaction between RNF168 and LSD1 in the chromatin-enriched fraction. 293T cells were transiently co-transfected with FLAG-RNF168 and HA-Vec/HA-LSD1. Co-transfectants with FLAG-RNF168 and HA-LSD1 were either mock-treated or treated with bleomycin (10 μg/ml) for different time points as indicated before harvest. Chromatin-enriched fractions (P3) were prepared 48 h after transfection and subjected to immunoprecipitation with an anti-HA antibody followed by immunoblotting with antibodies as indicated.

It has been demonstrated that LSD1 is recruited to DNA damage sites in an RNF168-dependent manner (23). The N-terminus of LSD1 (1–172 AAs) is required for its binding with RNF168. We thus sought to determine if CK2/WIP1-modulated LSD1 phosphorylation is important for its binding to RNF168. As shown in Figure 4A, when bacterially produced GST-LSD1 and HIS-RNF168 were mixed together, GST-LSD1 specifically pulled down HIS-RNF168 (Figure 4A). This indicated that LSD1 directly interacts with RNF168.

We next determined if CK2-mediated phosphorylation and WIP1-mediated dephosphorylation of LSD1 have an impact on the interaction between LSD1 and RNF168. We found that when bacterially produced GST-LSD1 was phosphorylated by CK2a’ in an *in vitro* kinase reaction, it pulled down more HIS-RNF168 than that without pre-phosphorylation by CK2a’, whereas this enhanced pulldown was diminished when TBB, a specific inhibitor of CK2, was present in the kinase reaction (Figure 4B). These results demonstrated that CK2-mediated phosphorylation promoted the interaction between LSD1 and RNF168. This conclusion was further supported by the following *in vivo* evidences: (i) the phosphorylation-defective mutants (HA-LSD1(S131A), HA-LSD1(S137A) and HA-LSD1(2A)) exhibited lower binding affinity with FLAG-RNF168 in 293T cells in the co-immunoprecipitation assays (Figure 4C and Supplementary Figure S4A); (ii) when cells were treated with TBB, both LSD1 phosphorylation at S131 and the interaction between LSD1 and RNF168 decreased in a dose-dependent manner (Figure 4D); (iii) expression of wild-type WIP1, but not the phosphatase-deficient mutant WIP1 (D314A), greatly reduced the interaction between LSD1 and RNF168 (Supplementary Figure S4B). It was also noted that the catalytically inactive mutant LSD1(K661A), like the wild-type LSD1, was able to interact with RNF168 without obvious difference (Supplementary Figure S4C), indicating that the demethylase activity of LSD1 is not essential for its binding to RNF168.

We then wanted to know if DNA damage induces LSD1 phosphorylation at S131 and S137. As shown in Figure 4E and Supplementary Figure S4D, both epitope-tagged and endogenous LSD1 phosphorylation at S131 and S137 in the chromatin-enriched fraction in 293T cells were detected at basal levels under unperturbed conditions and increased at 1 h after bleomycin treatment. Concomitantly, the levels of LSD1 present in the RNF168 immunocomplex gradually increased in the chromatin-enriched fraction within the first 2 h after bleomycin treatment (Figure 4F). These results demonstrated that DNA damage increased phosphorylation of LSD1 at S131 and S137 in chromatin.

### RNF168 promotes the interaction between LSD1 and 53BP1

**Figure 5.**
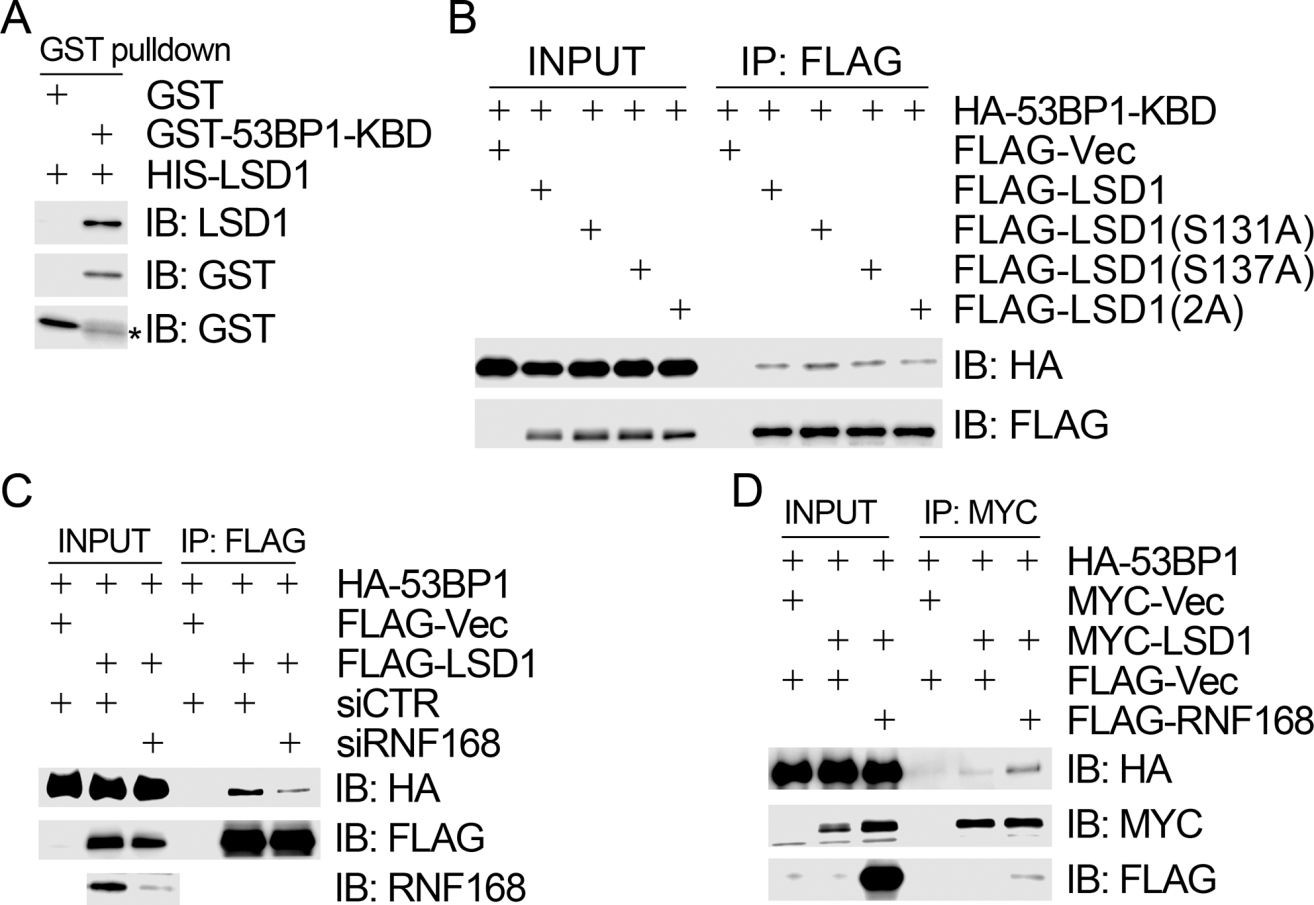
RNF168 promotes the interaction between 53BP1 and LSD1. (**A**) LSD1 interacted directly with 53BP1. Bacterially produced GST-53BP1-KBD was used to pull down bacterially produced HIS-LSD1 *in vitro*. (**B**) Phosphorylation-defective LSD1 mutants retained their interaction capacity with 53BP1. 293T cells were co-transfected with HA-53BP1-KBD and FLAG-Vec/FLAG-LSD1/HA-LSD1(S131A)/FLAG-LSD1(S137A)/FLAG-LSD1(2A). Total cell lysates were extracted 48 h after transfection and subject to immunoprecipitation with an anti-FLAG antibody followed by immunoblotting with antibodies as indicated. (**C**) Inhibition of RNF168 expression compromised the interaction between 53BP1 and LSD1. 293T cells were transfected first with siCTR or siRNF168 and then with HA-53BP1 and FLAG-Vec/FLAG-LSD1 24 h after the first transfection. Total cell lysates were harvested 48 h after the second transfection and subjected to immunoprecipitation with an anti-FLAG antibody followed by immunoblotting with antibodies as indicated. (**D**) Overexpression of RNF168 enhanced the interaction between 53BP1 and LSD1. 293T cells were co-transfected triply with HA-53BP1, MYC-Vec/MYC-LSD1 and FLAG-Vec/FLAG-RNF168. Total cell lysates were extracted 48 h after transfection and subjected to immunoprecipitation with an anti-MYC antibody followed by immunoblotting with antibodies as indicated.

It has been demonstrated that RNF168 interacts with 53BP1 and ubiquitinates its kinetochore-binding domain (KBD) (22). We thus sought to determine if LSD1 physically associates with 53BP1. We found that bacterially produced GST 53BP1-KBD was able to specifically pull down bacterially produced HIS-LSD1 (Figure 5A), indicating that LSD1 may directly interact with 53BP1. However, this interaction was not obviously modulated by LSD1 phosphorylation at S131 or S137 because the phosphorylation-defective mutants FLAG-LSD1(S131A), FLAG-LSD1(S137A) and FLAG-LSD1(2A) retained the same binding capacity to HA-53BP1-KBD as the wild-type FLAG-LSD1 in 293T cells (Figure 5B). We then attempted to determine if RNF168 regulates the interaction between LSD1 and 53BP1. As shown in Figure 5C, inhibition of RNF168 expression by siRNA compromised the interaction between FLAG-LSD1 and HA-53BP1 in 293T cells in the co-immunoprecipitation assays. Conversely, ectopic expression of FLAG-RNF168 increased the interaction between MYC-LSD1 and HA-53BP1 in 293T cells (Figure 5D). Taken together, these data demonstrated that CK2-mediated phosphorylation of LSD1 does not have an impact on its interaction with 53BP1, while RNF168 promotes the interaction between LSD1 and 53BP1.

### CK2-mediated phosphorylation of LSD1 promotes RNF168-dependent 53BP1 ubiquitination and recruitment in response to DNA damage

**Figure 6.**
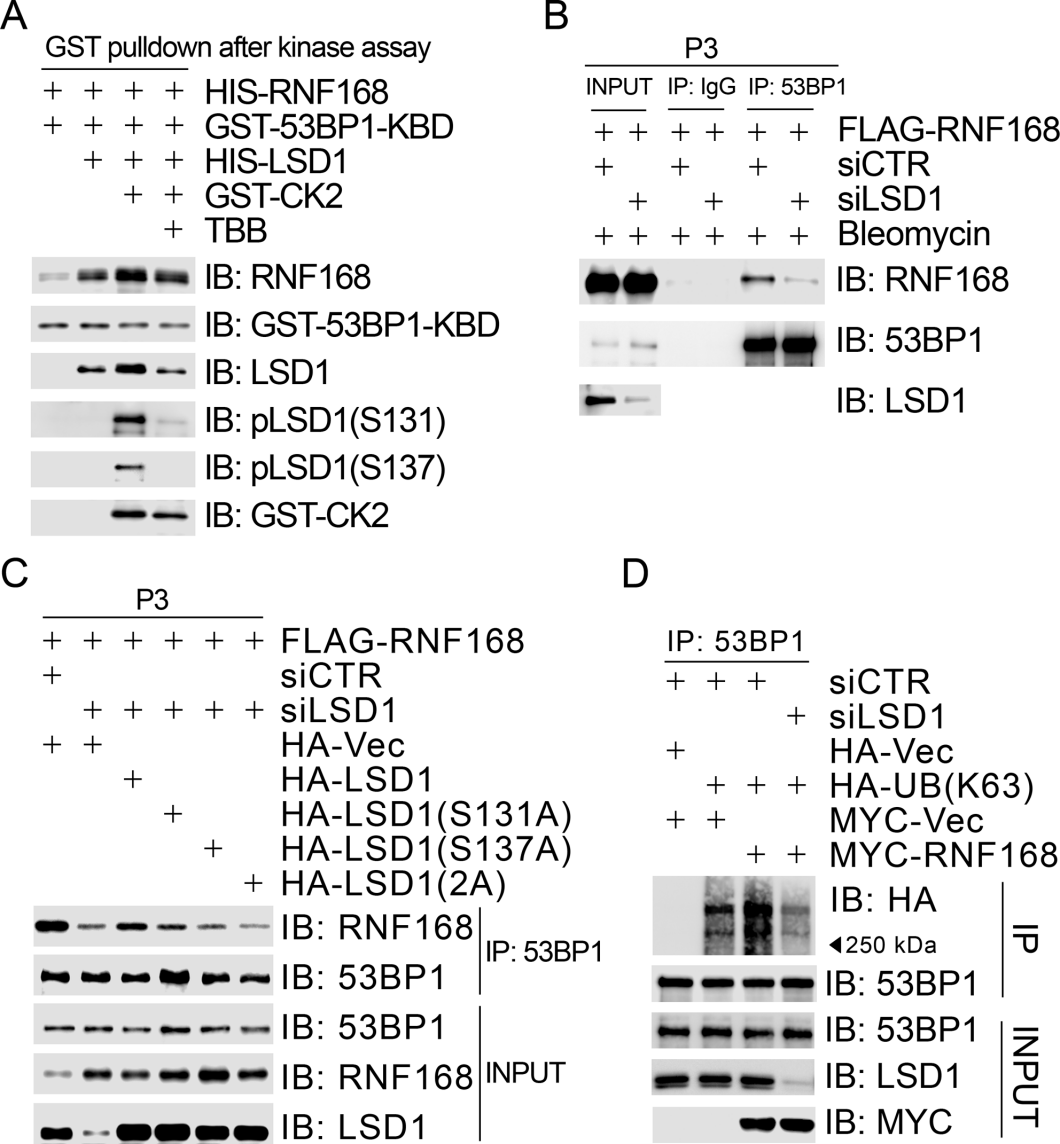
LSD1 phosphorylation promotes the interaction between 53BP1 and RNF168 in chromatin. (**A**) LSD1 interacted directly with 53BP1 and CK2-mediated phosphorylation promoted this interaction. Bacterially produced GST-53BP1-KBD was used to pull down bacterially produced HIS-RNF168 in the absence of HIS-LSD1 or in the presence of HIS-LSD1, HIS-LSD1 phosphorylated by CK2α’ *in vitro* in the absence/presence of the CK2 inhibitor TBB. (**B**) Inhibition of LSD1 expression compromised the interaction between 53BP1 and RNF168 in the chromatin-enriched fraction. 293T cells were transfected first with siCTR or siLSD1 and then with FLAG-RNF168 24 h after the first transfection. Cells were treated with bleomycin (10 μg/ml) for 1 h before harvest. Chromatin-enriched fractions (P3) were extracted 48 h after the second transfection and subjected to immunoprecipitation followed by immunoblotting with antibodies as indicated. (**C**) Phosphorylation-defective LSD1 mutants reduced the interaction between 53BP1 and RNF168. 293T cells were transfected first with siCTR or siLSD1 and then co-transfected with FLAG-RNF168 and HA-Vec/HA-LSD1/HA-LSD1(S131A)/HA-LSD1(S137A)/HA-LSD1(2A) 24 h after the first transfection. Chromatin-enriched fractions (P3) were extracted 48 h after the second transfection and subjected to immunoprecipitation with an anti-53BP1 antibody followed by immunoblotting with antibodies as indicated. (**D**) Inhibition of LSD1 expression compromised RNF168-mediated 53BP1 ubiquitination. 293T cells were transfected first with siCTR or siLSD1 and then co-transfected with HA-Vec/HA-UB(K63) and MYC-Vec/MYC-RNF168 24 h after the first transfection. Total cell lysates were extracted 48 h after the second transfection and subjected to immunoprecipitation with an anti-53BP1 antibody followed by immunoblotting with antibodies as indicated.

Hekem *et al*. recently uncovered that RNF168-mediated K63-linked polyubiquitination of 53BP1 is essential for the initial recruitment of 53BP1 to the DNA damage site (22). Given that CK2-mediated phosphorylation of LSD1 promotes its binding to RNF168 (Figure 4) and that RNF168 promotes the binding of LSD1 to 53BP1, it was tempting to determine if CK2-mediated LSD1 phosphorylation modulates RNF168-dependent 53BP1 ubiquitination and recruitment to the DNA damage site. We found that bacterially produced GST-53BP1-KBD was able to specifically pull down bacterially produced HIS-RNF168, and this pulldown effect was enhanced in the presence of HIS-LSD1, and further enhanced when HIS-LSD1 was pre-incubated with CK2 in an *in vitro* kinase reaction, whereas this further enhancement was diminished when TBB was present in the *in vitro* kinase reaction (Figure 6A). These results suggested that CK2-mediated LSD1 phosphorylation promotes the interaction between RNF168 and 53BP1. This conclusion was further supported by *in vivo* evidences: (i) inhibition of LSD1 expression compromised the interaction between FLAG-RNF168 and the endogenous 53BP1 (Figure 6B) and (ii) LSD1 depletion-induced decrease of the interaction between the endogenous 53BP1 and FLAG-RNF168 was corrected by re-introduction of siRNA-resistant wild-type HA-LSD1, but not the phosphorylation-defective mutant HA-LSD1(S131A), HA-LSD1(S137A) or HA-LSD1(2A) (Figure 6C). As shown in Figure 6D, we further found that expression of ectopic MYC-RNF168 increased K63-linked polyubiquitination of the endogenous 53BP1 in 293T cells, whereas inhibition of LSD1 expression by siRNA lowered the 53BP1 ubiquitination levels to an extent less than that in the mocked depletion, even in the presence of MYC-RNF168.

**Figure 7.**
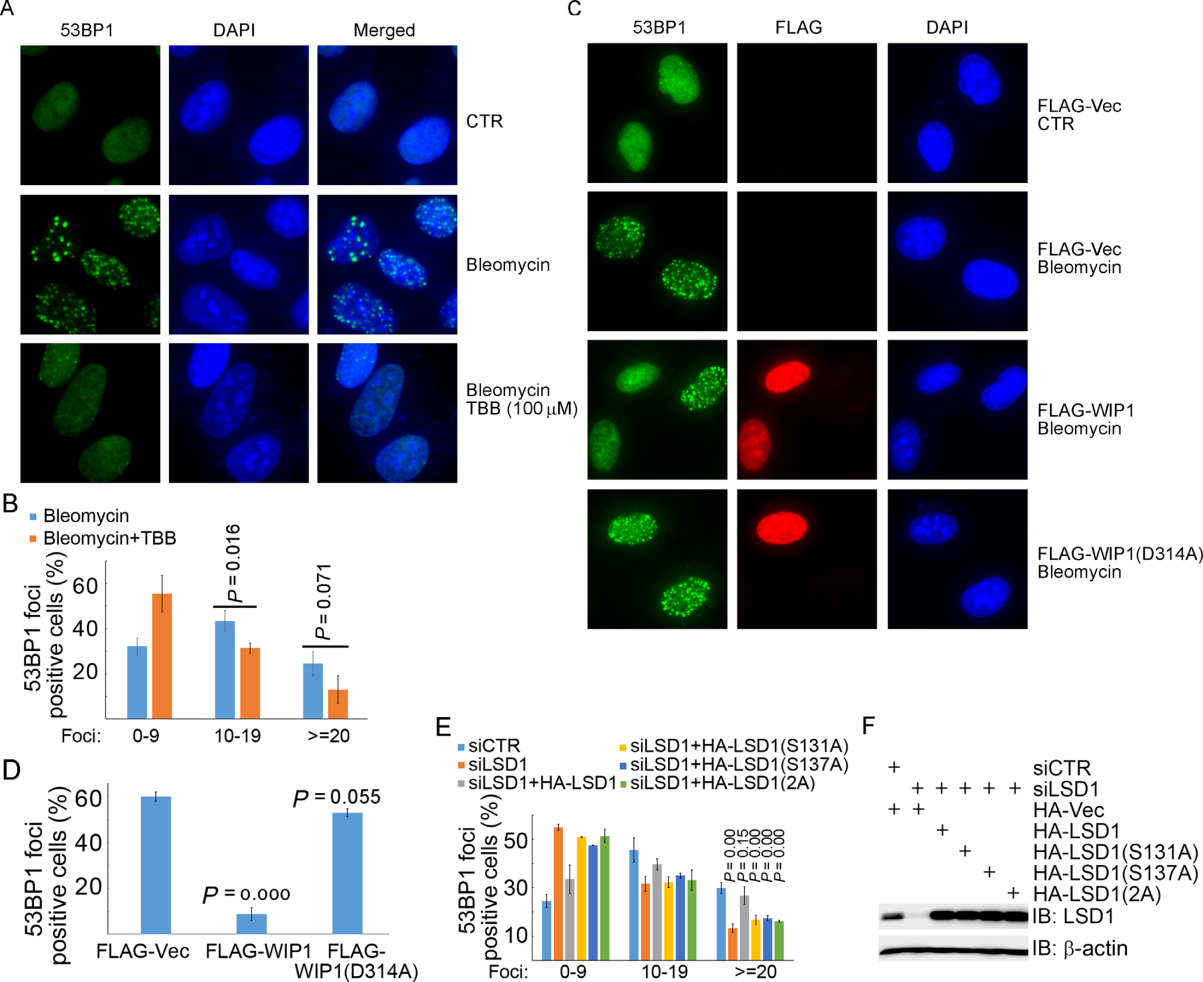
LSD1 phosphorylation promotes 53BP1 recruitment to the DNA damage sites. (**A** and **B**) Inhibition of CK2 activity compromised bleomycin-induced 53BP1 recruitment. U2OS cells were either untreated or treated with bleomycin (10 μg/ml) for 1 h without or with pretreatment with TBB (100 μM) for 6 h. Cells were fixed and proceeded for immunofluorescence (IF) staining with an anti-53BP1 antibody. Representative images are shown in (A) and the quantitation data of 53BP1 foci per cell is in (B). (**C** and **D**) Overexpression of WIP1 compromised 53BP1 recruitment. U2OS cells were transfected with FALG-Vec, FLAG-WIP1 or FLAG-WIP1 (D314A). Transfectants were either untreated or treated with bleomycin (10 μg/ml) for 1 h before IF. IF was performed 48 h after transfection with antibodies as indicated. Representative images are shown in (C) and the quantitation data of > = 10 53BP1 foci per cell is in (D). (**E** and **F**) Defective recruitment of 53BP1 to the DNA damage sites induced by inhibition of LSD1 expression was rescued by re-expression of the wild-type LSD1, but not the phosphorylation-defective LSD1 mutants. U2OS cells were engineered to stably express HA-Vec, HA-LSD1, HA-LSD1(S131A), HA-LSD1(S137A) or HA-LSD1(2A) by retroviral infection. Infectants were transfected with siCTR or siLSD1. Transfectants were synchronized by double thymidine blocks 6 h after transfection and released for 6 h. Cells were then either untreated or treated with bleomycin (10 μg/ml) for 1 h. IF was performed with antibodies as indicated. Representative images are shown in Supplementary Figure S5, while the quantitation data of 53BP1 foci per cell is in (E), and immunoblotting data is in (F).

It has been demonstrated that inhibition of LSD1 expression by shRNA reduces 53BP1 recruitment at DSB sites (23). We found that inhibition of CK2 kinase activity by TBB in U2OS cells reduced bleomycin-induced 53BP1 enrichment on the DNA damage sites (10–19 or > = 20 foci per cell) (Figure 7A and B). Transient expression of wild-type WIP1, but not the phosphatase-deficient mutant WIP1 (D314A) in U2OS cells, diminished bleomycin-induced 53BP1 recruitment (Figure 7C and D). Furthermore, inhibition of LSD1 expression by siRNA reduced 53BP1 recruitment (> = 20 foci per cell), and this reduction was corrected by re-introduction of wild-type LSD1, but not by re-introduction of LSD1(S131A), LSD1(S137A) or LSD1(2A) in U2OS cells (Figure 7E, F, and Supplementary Figure S5). Taken together, our results demonstrated that CK2-mediated phosphorylation of LSD1 promotes RNF168-dependent 53BP1 ubiquitination and recruitment in response to DNA damage.

### Phosphorylation of LSD1 at S131 and S137 promotes cell proliferation and survival in response to genotoxic stress

**Figure 8.**
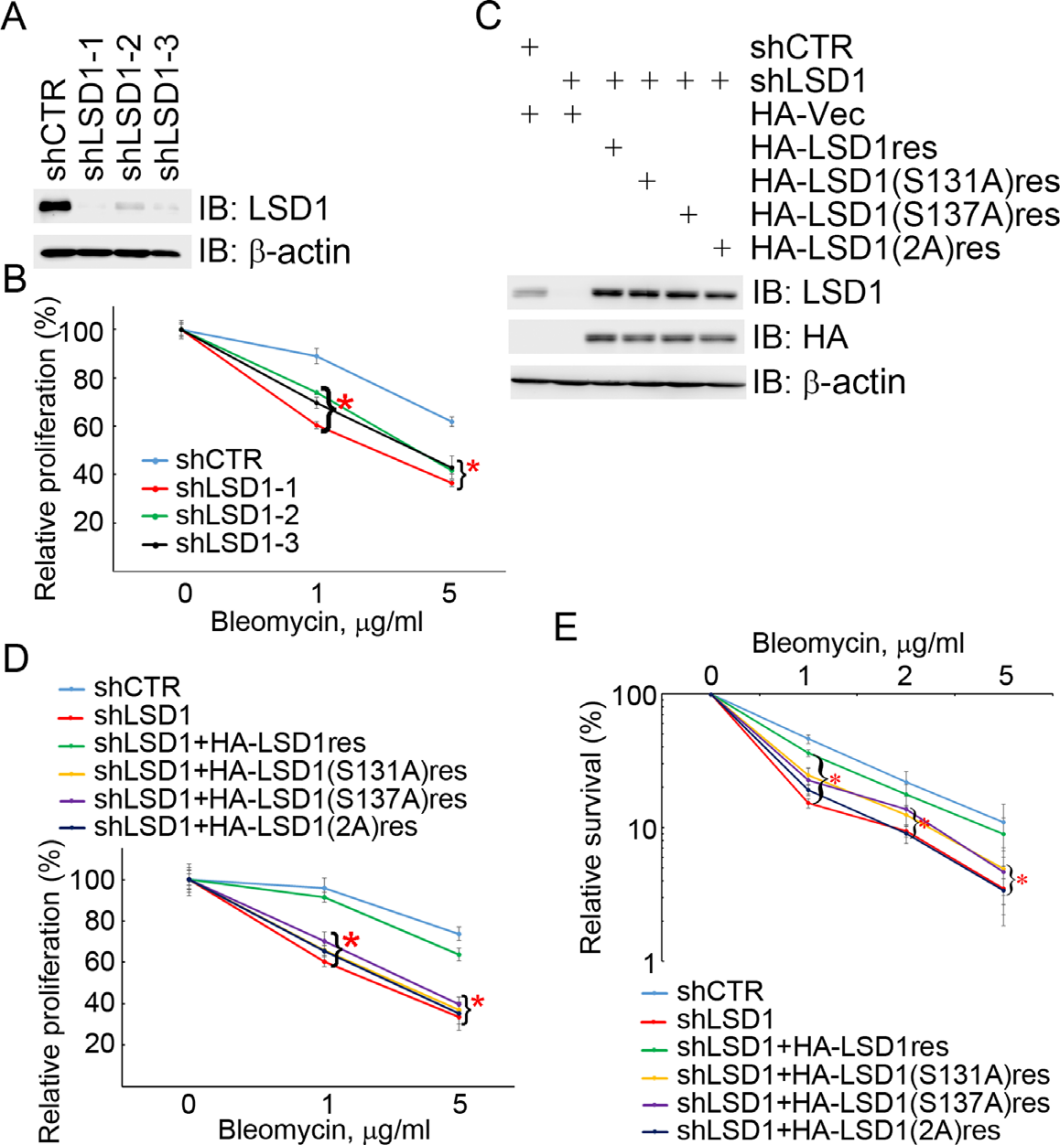
LSD1 phosphorylation promotes cell proliferation and survival in response to DNA damage. (**A** and **B**) Inhibition of LSD1 expression sensitized cells to bleomycin treatment. HEK293 cells were stably infected with lentiviral shCTR, shLSD1–1, shLSD1–2 or shLSD1–3. Total cell lysates were extracted and subjected to immunoblotting with antibodies as indicated in (A). Infectants were treated with bleomycin with increasing concentrations for 72 h. Relative cell proliferation was determined by MTT assay (B). (**C–E**) Phosphorylation-defective LSD1 mutants failed to correct LSD1 depletion-induced cellular sensitization to bleomycin treatment. The shLSD1–1-mediated stable knockdown HEK293 cells described in (A) were engineered to stably express HA-Vec, HA-LSD1res, HA-LSD1(S131A)res, HA-LSD1(S137A)res or HA-LSD1(2A)res by retroviral infection. Infectants were treated with bleomycin with increasing concentrations for 72 h. LSD1 protein levels were shown in (C), relative cell proliferation was determined by MTT assay (D) and relative cell survival was measured by clonogenic assay (E). *P < 0.05.

To examine if LSD1 phosphorylation at S131 and S137 plays a role in cell proliferation and survival in response to DNA damage, we generated HEK293 cells with stable knockdown of LSD1 expression by lentiviral infection with three specific shRNAs (Figure 8A). Inhibition of LSD1 expression compromised cellular proliferation after bleomycin treatment in a dose-dependent manner (Figure 8B). To exclude the possibility of shRNA off-target effects, we re-introduced the wild-type HA-LSD1 or the phosphorylation-defective mutant HA-LSD1(S131A), HA-LSD1(S137A) or HA-LSD1(2A), all of which were insensitive to the inhibition by shLSD1–1, into the HEK293 cells by retroviral infection, in which the endogenous LSD1 was stably knocked down by shLSD1–1 (Figure 8C). We found that re-expression of the wild-type HA-LSD1res, but not the phosphorylation mutants, partially corrected LSD1 depletion-induced sensitization to bleomycin or etoposide treatment in HEK293 cells in a cell proliferation assay (Figure 8D and Supplementary Figure S6) and a cell survival assay (Figure 8E). These results demonstrated that phosphorylation of LSD1 at S131 and S137 promotes cell proliferation and survival in response to genotoxic stress.

## DISCUSSION

**Figure 9.**
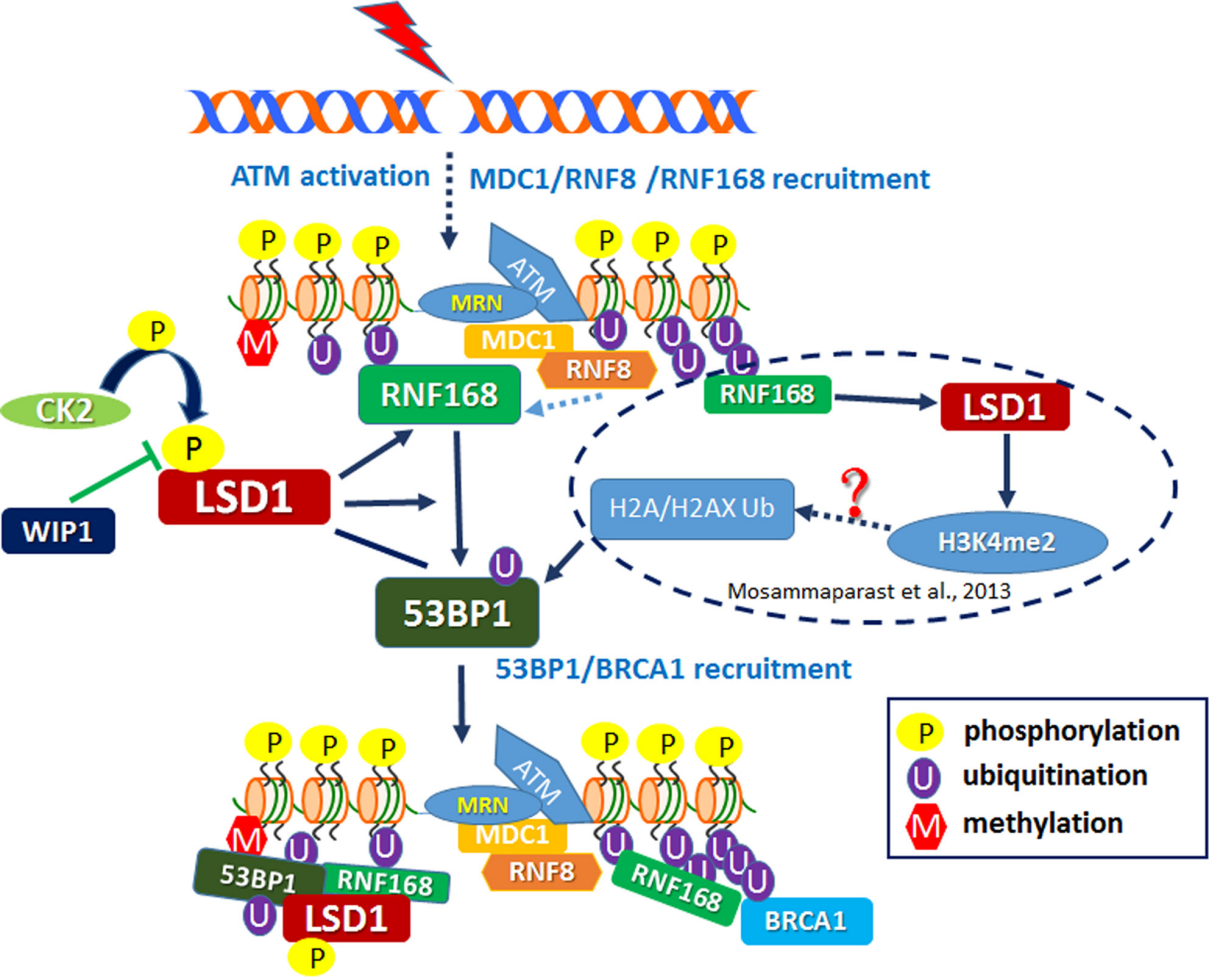
A working model showing that CK2-mediated phosphorylation of LSD1 at S131 and S137 promotes RNF168-dependent recruitment of 53BP1 to the sites of DNA damage. A solid arrow depicts a positive regulation of the particular process or promotes the interaction between the two proteins, while a dotted arrow indicates a multiple-step process, and a solid line represents a constitutive or unregulated interaction between the two proteins. Details of this model are described in the Discussion section.

Initial recruitment and retention of 53BP1 at the DNA damage site is a complicated process regulated by mechanisms with a common goal, in which H4K20 is properly methylated and accessible by 53BP1. A group of methyltransferases, including SET8 and MMSET, and certain demethylase(s) unidentified yet act together to maintain H4K20me2 status, while other mediators work to unmask H4K20me2 by removal of occupiers of H4K20me2 under unperturbed conditions and/or to remodel chromatin locally for the exposure of H4K20me2 (15,16). Our results demonstrated that CK2-mediated phosphorylation of LSD1 promotes the direct interaction between RNF168 and 53BP1 and RNF168-dependent ubiquitination of 53BP1, presumably bringing 53BP1 to a closed juxtaposition to H4K20me2. Given that CK2-mediated phosphorylation of LSD1 increases its interaction with RNF168, it is possible that local enrichment of LSD1 may promote H3K4me2 demethylation and may subsequently enhance H2A/H2AX ubiquitination, which would be consistent with Mosammaparast *et al*.'s observation (23). However, the cross-talk between H3K4me2 and H2A/H2AX ubiquitination has not been well documented yet. The simplest explanation is that LSD1-mediated demethylation of H3K4 makes local chromatin more accessible for RNF8/RNF168, the E3 ligases for H2A/H2AX ubiquitination in response to DNA damage. Indeed, it was observed by immunofluorescence staining that ionizing radiation (IR)-induced γ-H2AX foci lacked H3K4me staining (46). Nevertheless, we could not exclude the possibility that recruitment of LSD1 to the DNA damage sites may directly promote RNF8/RNF168-mediated H2A/H2AX ubiquitination, while local demethylation of H3K4 simply is resulted from local enrichment of LSD1 and may not contribute much to H2A/H2AX ubiquitination. We thus speculate that both demethylase-dependent and demethylase-independent mechanisms may work together to accomplish LSD1-mediated 53BP1 recruitment (Figure 9).

The N-terminus of LSD1 protein is very flexible and thus its structure has not been solved yet. It has been demonstrated to be dispensable for its demethylase activity (44,47). However, it is rich in Ser/Thr residues, suggesting that phosphorylation of this region may modulate unknown functions of LSD1. It was found that deletion of the N-terminus of LSD1 abolished its association with RNF168 and its recruitment to laser-induced DNA damage sites (23). Inhibition of CK2 expression compromised 53BP1 recruitment to the DNA damage sites (31). Our results uncovered that CK2 phosphorylates LSD1 at S131 and S137 both *in vitro* and *in vivo*, while WIP1 dephosphorylates LSD1 at S131 and S137 both *in vitro* and *in vivo*. CK2-mediated phosphorylation of LSD1 increases its binding to RNF168, the interaction between RNF168 and 53BP1, and RNF168-dependent K63-linked polyubiquitination of 53BP1, promoting direct recruitment of both LSD1 and 53BP1 to the DNA damage site (Figure 9). This adds another layer of regulatory complexity of RNF168-dependent and LSD1-facilitated recruitment of 53BP1 to the DNA damage site (Figure 9). However, how CK2-mediated phosphorylation of LSD1 promotes its binding to RNF168 is still illusive. RNF168 has three ubiquitin-binding domains (48) though it does not possess any obvious phosphorylation binding module. It has been reported recently that LSD1 is ubiquitinated and targeted for proteasome-mediated degradation (49). Two proteome-wide screens (50,51) identified at least six ubiquitination sites within the second coiled-coil domain of LSD1 (aa 428–514). We thus speculate that CK2-mediated phosphorylation of LSD1 may be beneficial for LSD1 ubiquitination by RNF168 or another unknown E3 ligase.

It is noted that the CK2-specific inhibitor TBB only partially compromised microirradiation-induced LSD1 recruitment, while most of the phosphorylation-defective mutants LSD1(S131A) and LSD1(S137A) were not able to be recruited to sites of DNA damage (Figure 3). This suggests that additional kinase(s)-mediated phosphorylation of LSD1 at S131 and S137 may play a role in DNA damage-induced recruitment of LSD1, which warrants further investigation.

Given that CK2 is a constitutive active kinase with more than 300 substrates identified, by far there is no obvious evidence that DNA damage induces CK2 enzymatic activity. Nevertheless, recruitment of CK2 to the DNA damage sites (29) may promote phosphorylation of a variety of DDR factors, including MDC1. Thus, we believe that DDR may modulate CK2 localization/recruitment instead of its enzymatic activity.

LSD1 has been found to be overexpressed in a variety of cancers, including breast cancer, liver cancer and tongue cancer, and its expression status is correlated with its oncogenic effects in tumorigenesis (52–54). Silencing of the mismatch repair gene *MLH1* is frequently seen in sporadic colon cancers. This silencing is largely achieved through the LSD1/CoREST complex-mediated demethylation of H3K4 at the *MLH1* promoter (55). Mutations within the N-terminus of LSD1 have been found in different cancers, including A85V in 1 out of 56 cases of nasopharyngeal carcinoma (56), P152T in 1 out of 489 cases of ovarian serous adenocarcinoma (57) and S172A in 1 case of colorectal adenocarcinoma (58). In this report, we revealed the biological significance of CK2-mediated phosphorylation of LSD1 at S131 and S137, which promotes cell proliferation and survival after DNA damage. Taken together, our results along with discoveries by other groups have demonstrated that inhibition of LSD1 expression and/or post-translational modification may be a potentially promising approach for improving cancer therapy with DNA damaging agents, for example as ionizing radiation.

## SUPPLEMENTARY DATA

Supplementary Data are available at NAR Online.

SUPPLEMENTARY DATA
