# Supplementary material for: Modulation of LSD1 phosphorylation by CK2/WIP1 regulates RNF168-dependent 53BP1 recruitment in response to DNA damage
Source: Nucleic Acids Res. 2015 May 20;43(12):5936–47. doi: 10.1093/nar/gkv528 (PMC4499147; doi:10.1093/nar/gkv528)
Supplement: SUPPLEMENTARY DATA [file supp_43_12_5936__index.html]

Modulation of LSD1 phosphorylation by CK2/WIP1 regulates RNF168-dependent 53BP1 recruitment in response to DNA damage — Modulation of LSD1 phosphorylation by CK2/WIP1 regulates RNF168-dependent 53BP1 recruitment in response to DNA damage — SUPPLEMENTARY DATA 

# Modulation of LSD1 phosphorylation by CK2/WIP1 regulates RNF168-dependent 53BP1 recruitment in response to DNA damage

## SUPPLEMENTARY DATA

- SUPPLEMENTARY DATA
