# Supplementary material for: Modulation of LSD1 phosphorylation by CK2/WIP1 regulates RNF168-dependent 53BP1 recruitment in response to DNA damage
Source: Nucleic Acids Res. 2015 May 20;43(12):5936–47. doi: 10.1093/nar/gkv528 (PMC4499147; doi:10.1093/nar/gkv528)
Supplement: SUPPLEMENTARY DATA [file supp_gkv528_nar-00497-d-2015-File011.pdf]

Peng et al., supplemental figures

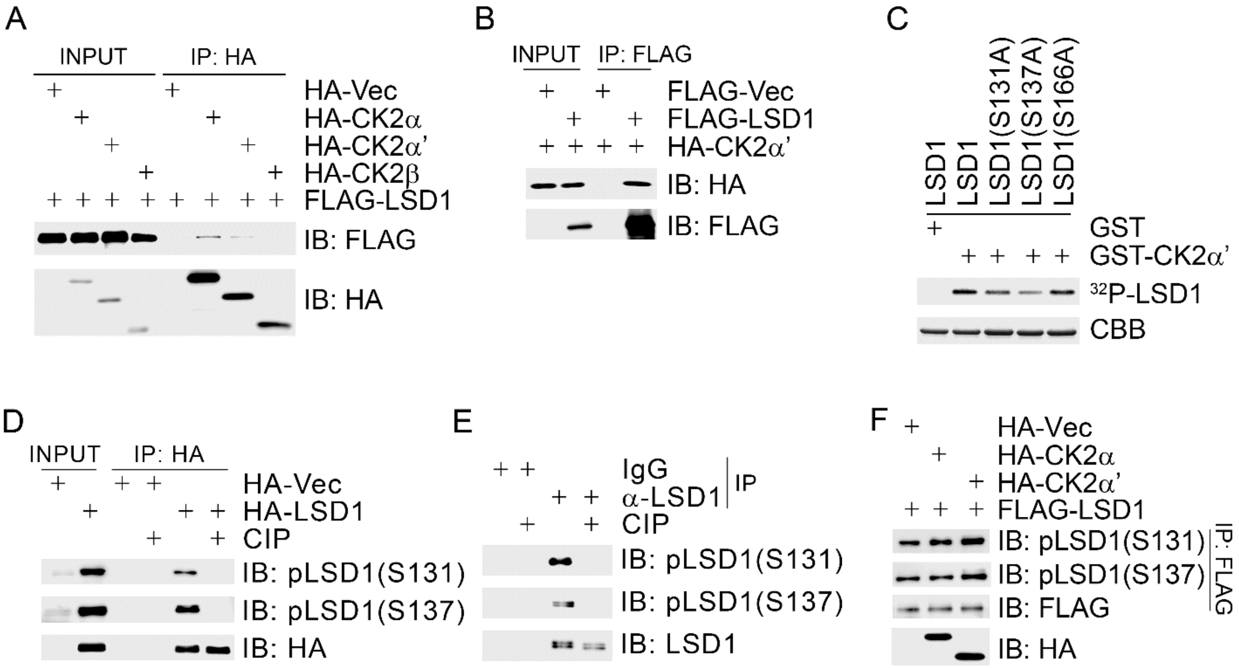

Figure S1. CK2 phosphorylates LSD1 at S131 and S137 both in vitro and in vivo. (A) FLAG-LSD1 was present in the HA-CK2 immunocomplex. Total cell lysates were extracted from 293T cells transiently co-transfected with FLAG-LSD1 and HA-Vec/HA-CK2α/HA-CK2α'/HA-CK2β and subjected to immunoprecipitation with an anti-HA antibody followed by immunoblotting with antibodies as indicated. (B) HA-CK2 was present in the FLAG-LSD1 immunocomplex. Total cell lysates were extracted from 293T cells transiently co-transfected with HA-CK2α' and FLAG-Vec/FLAG-LSD1 and subjected to immunoprecipitation with an anti-FLAG antibody followed by immunoblotting with antibodies as indicated. (C) S166 is not a major phosphorylation site of LSD1 by CK2 in vitro. GST or GST- CK2α' recombinant protein was incubated with bacterially produced HIS-LSD1 or its phosphorylation-defective mutants in the presence of ^{32}P-γATP in the in vitro kinase assay. (D and E) The immunoreactive signal of the LSD1 phospho-specific antibodies was diminished upon CIP treatment. The immunocomplexes of HA-LSD1 (D) or endogenous LSD1 (E) from 293T cells with mocked or CIP treatment were subjected

immunoblotting with antibodies as indicated. (F) Over-expression of CK2 slightly enhanced LSD1 phosphorylation at S131 and S137. 293T cells were transiently transfected with expression constructs as indicated. Total cell lysates were harvested 48 hours after transfection and subjected to immunoprecipitation with an anti-FLAG antibody followed by immunoblotting with antibodies as indicated.

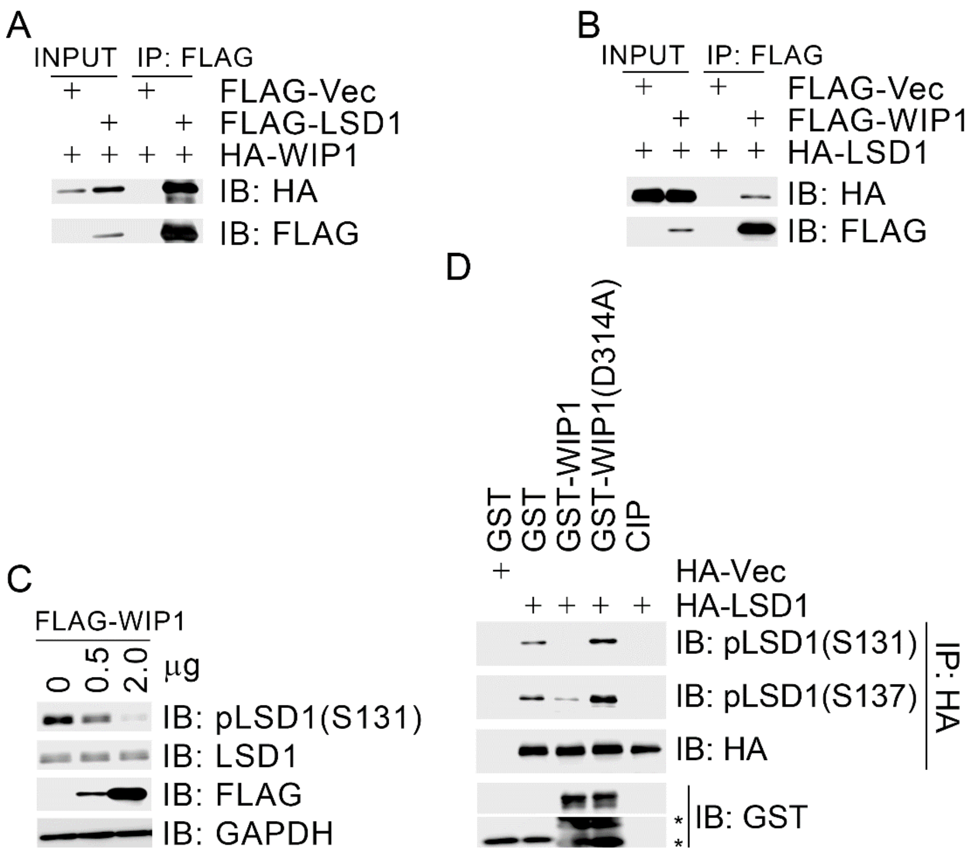

Figure S2. WIP1 dephosphorylates LSD1 at S131 and S137 both in vitro and in vivo. (A and B) Epitope-tagged WIP1 co-immunoprecipitated reciprocally with epitope-tagged LSD1. Total cell lysates were extracted from 293T cells transiently cotransfected with expression constructs as indicated and subjected to immunoprecipitation followed by immunoblotting with antibodies as

indicated. (C) Overexpression of WIP1 decreased LSD1 phosphorylation at S131 in a dose-dependent manner in vivo. HeLa cells were transiently transfected with different amount of FLAG-WIP1. Total cell lysates were harvested 48 hours after transfection and subjected to immunoblotting with antibodies as indicated. (D) WIP1 dephosphorylated HA-LSD1 at S131 and S137 in vitro. CIP or bacterially produced GST, GST-WIP1, or GST-WIP1(D314A) was incubated with the HA-LSD1 immuno-complexes prepared from 293T cells in the in vitro dephosphorylation assay.

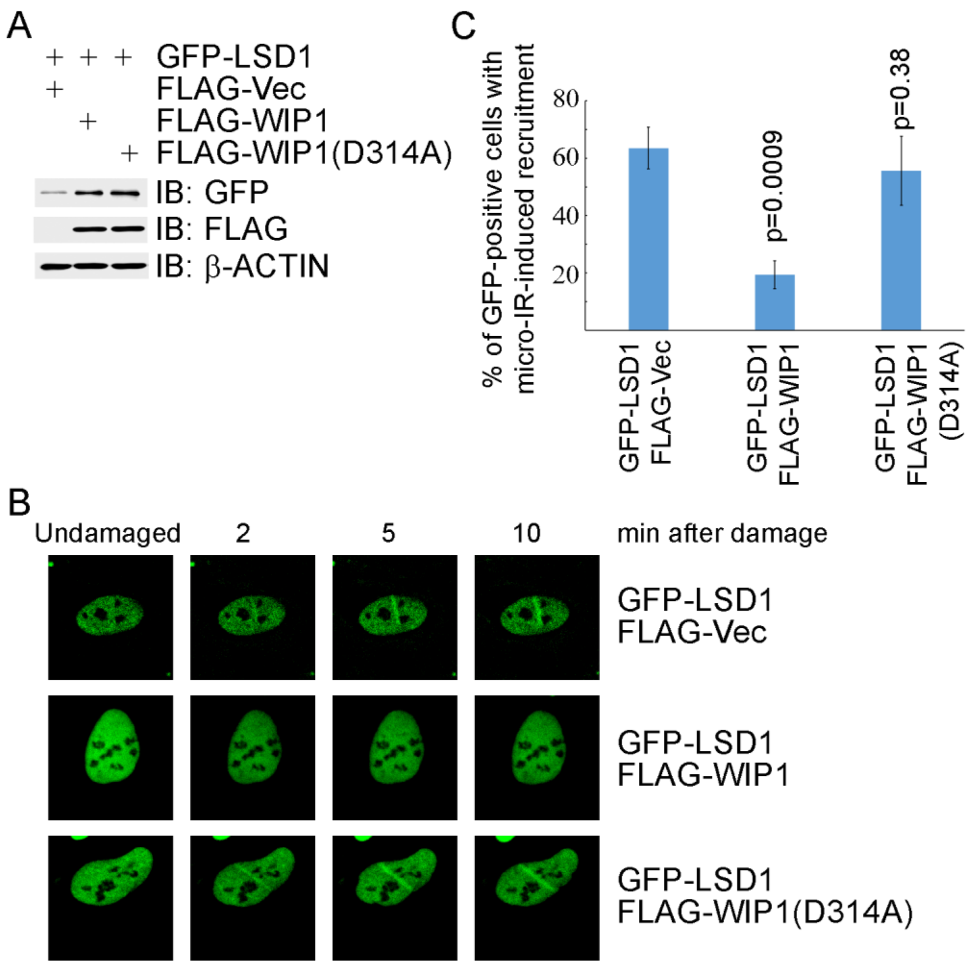

Figure S3. Overexpression of wild-type WIP1, but not the catalytically inactive mutant WIP1(D314A), compromised micro-irradiation-induced LSD1 recruitment. U2OS cells were co-transfected with GFP-LSD1 and FLAG-Vec, FLAG-WIP1, or FLAG-WIP1(D314A) (the mass ration was 1:10). Transfectants were irradiated 48 hours after transfection with a 365-nm UV laser beam. Images were collected every 60 seconds after irradiation. Total cell lysates were extracted after irradiation and subjected to immunoblotting with antibodies as indicated in (A). Representative images are shown in (B). In each set of experiment, more than 20 GFP-positive cells were irradiated and monitored for enrichment of GFP signal along the irradiation path. Three experimental repeats were performed and statistical analysis is shown in (C).

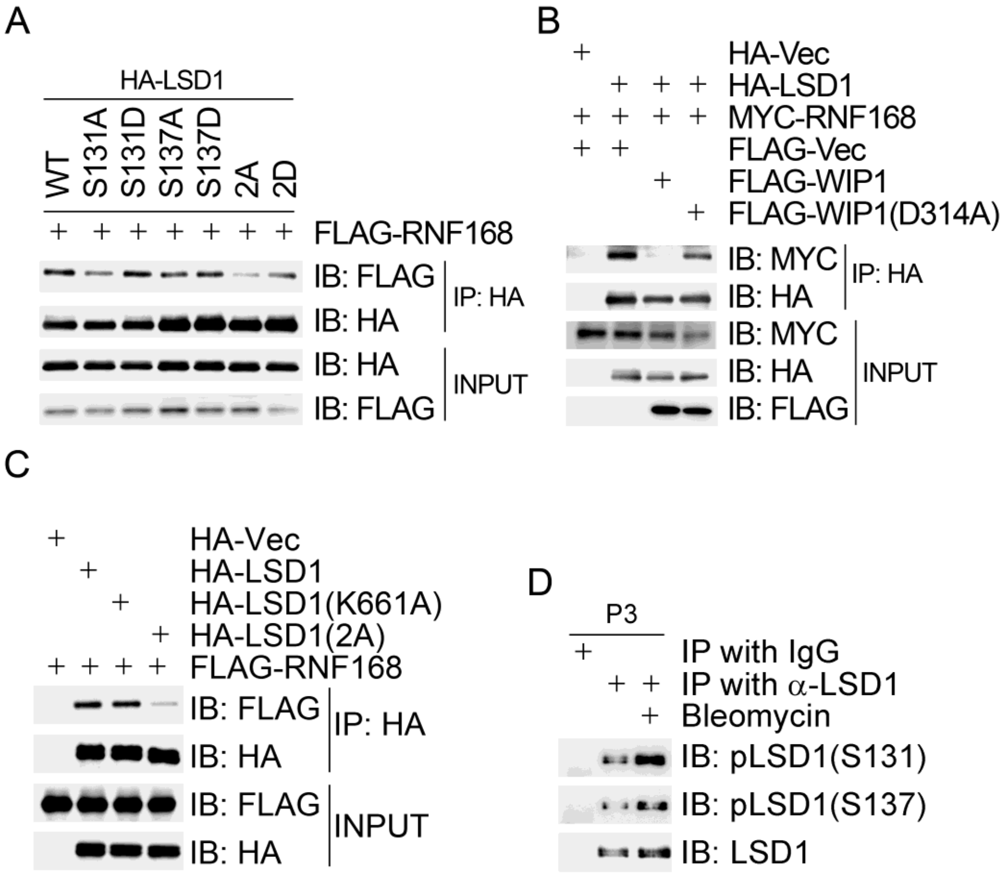

Figure S4. CK2-mediated phosphorylation of LSD1 promotes its association with RNF168. (A). Phosphorylation defective mutants LSD1(S131A), LSD1(S137A), and LSD1(2A) reduced their binding with RNF168. 293T cells were cotransfected with a phosphorylation-defective mutant (HA-LSD1(S131A), HA-LSD1(S137A), or HA-LSD1(2A)) or a phosphorylation-mimic mutant (HA-LSD1(S131D), HA-LSD1(S137D), or HA-LSD1(2D)) and FLAG-RNF168. Total cell lysates were extracted 48 hours after transfection and subjected to immunoprecipitation with an anti-HA antibody followed by immunoblotting with antibodies as indicated. (B). Overexpression of WIP1 blocked the interaction between RNF168 and LSD1. 293T cells were cotransfected triply with MYC-RNF168, HA-Vec/HA-LSD1, and FLAG-Vec/FLAG-WIP1/FLAG-WIP1(D314A). Total cell lysates were extracted 48 hours after transfection and subjected to immunoprecipitation with an anti-HA antibody followed by immunoblotting with antibodies as indicated. (C) The interaction of LSD1 and RNF168 is independent of the catalytic activity of LSD1. 293T cells were cotransfected with FLAG-RNF168 and HA-Vec/HA-LSD1/HA-LSD1(K661A)/HA-LSD1(2A). Total cell lysates were extracted 48 hours after transfection and subjected to immunoprecipitation with an anti-HA antibody followed by immunoblotting with antibodies as indicated. (D) DNA damage increased LSD1 phosphorylation in the chromatin-enriched fraction. 293T cells were either mock-treated or treated with bleomycin (10 μg/ml) for 1 hour before harvest. Chromatin-enriched fractions (P3) were prepared and subjected to immunoprecipitation with an anti-LSD1 antibody followed by immunoblotting with antibodies as indicated.

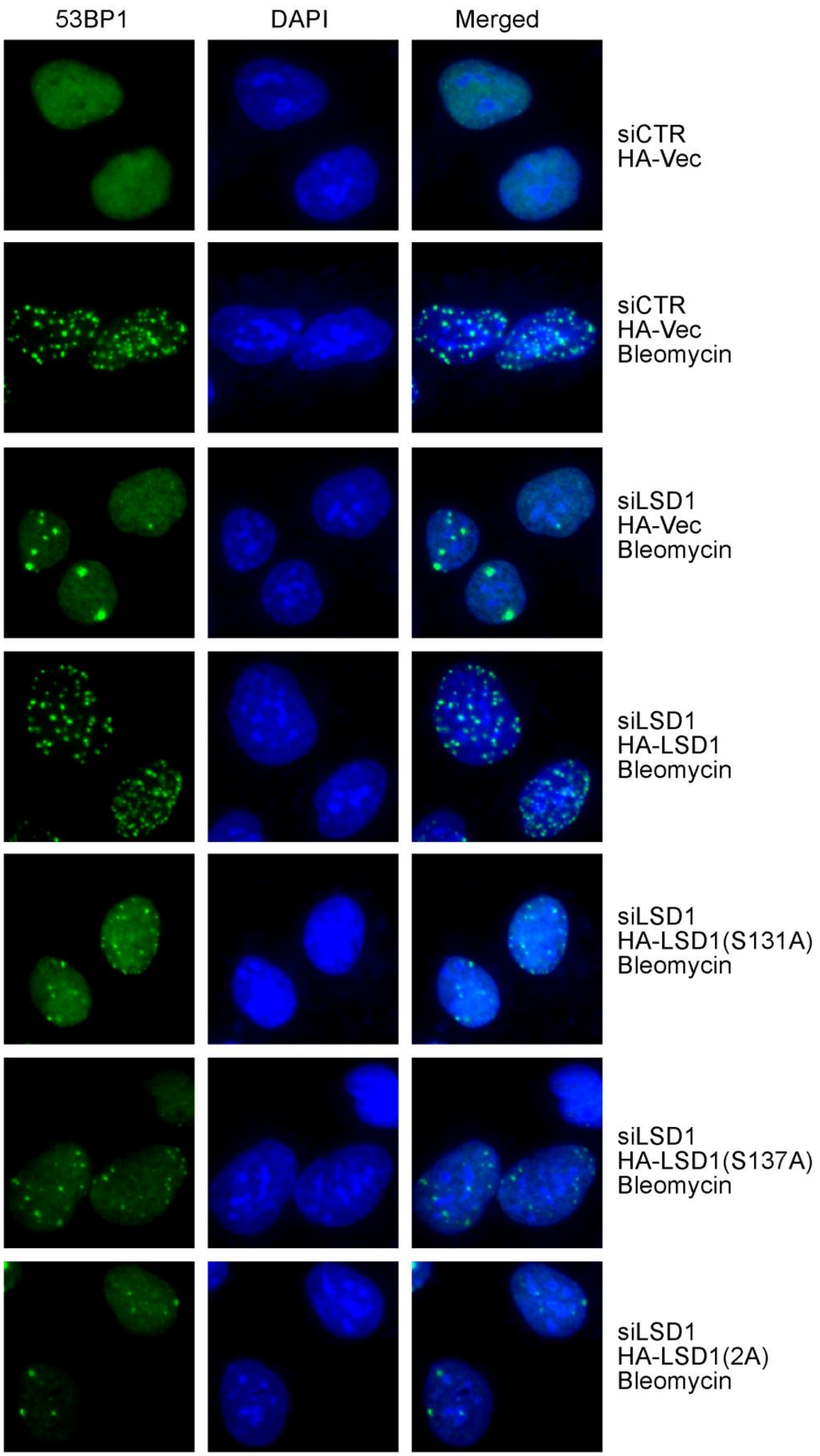

53BP1
DAPI
Merged
siCTR
HA-Vec
siCTR
HA-Vec
Bleomycin
siLSD1
HA-Vec
Bleomycin
siLSD1
HA-LSD1
Bleomycin
siLSD1
HA-LSD1(S131A)
Bleomycin
siLSD1
HA-LSD1(S137A)
Bleomycin
siLSD1
HA-LSD1(2A)
Bleomycin

Figure S5. Defective recruitment of 53BP1 to the DNA damage sites induced by inhibition of LSD1 expression was rescued by re-expression of the wild-type LSD1, but not the phosphorylation-defective LSD1 mutants. U2OS cells were engineered to stably express HA-Vec, HA-LSD1, HA-LSD1(S131A), HA-LSD1(S137A), or HA-LSD1(2A) by retroviral infection. Infectants were transfected with siCTR or siLSD1. Transfectants were synchronized by double thymidine blocks 6 hours after transfection and released for 6 hours. Cells were then either untreated or treated with bleomycin (10 μg/ml) for 1 hour. IF was performed with antibodies as indicated. Representative images are shown here, while the quantitation data of 53BP1 foci per cell is in Fig. 7E, and immunoblotting data is in Fig. 7F.

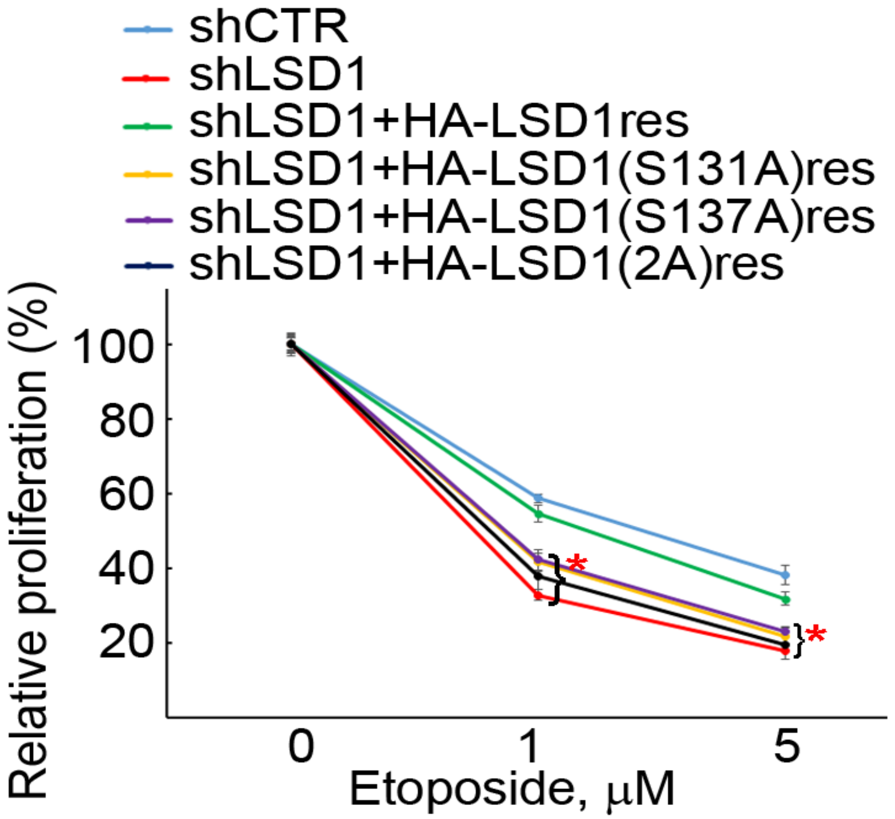

Figure S6. Phosphorylation-defective LSD1 mutants failed to correct LSD1 depletion-induced cellular sensitization to etoposide treatment. The shLSD1-1-mediated stable knockdown HEK293 cells described in Figure 8A were engineered to stably express HA-Vec, HA-LSD1res, HA-LSD1(S131A)res, HA-LSD1(S137A)res, or HA-LSD1(2A)res by retroviral infection. Infectants were treated with etoposide with increasing concentrations as indicated for 72 hours. Relative cell proliferation was determined by a MTT assay. *: $p<0.05$
